# Characterization and Study of Gene Expression Profiles of Human Periodontal Mesenchymal Stem Cells in Spheroid Cultures by Transcriptome Analysis

**DOI:** 10.1155/2021/5592804

**Published:** 2021-10-19

**Authors:** Takenori Suga, Michihiko Usui, Satoru Onizuka, Kotaro Sano, Tsuyoshi Sato, Kohji Nakazawa, Wataru Ariyoshi, Tatsuji Nishihara, Keisuke Nakashima

**Affiliations:** ^1^Division of Periodontology, Department of Oral Function, Kyushu Dental University, 2-6-1 Manazuru, Kokurakita-ku, Kitakyushu 803-8580, Japan; ^2^Department of Oral and Maxillofacial Surgery, Saitama Medical University, 38 Moro-hongou, Moroyama-machi, Iruma-gun, Saitama 350-0495, Japan; ^3^Department of Life and Environment Engineering, The University of Kitakyushu, 1-1 Hibikino, Wakamatsu-ku, Kitakyushu 808-0135, Japan; ^4^Division of Infection and Molecular Biology, Department of Health Improvement, Kyushu Dental University, 2-6-1 Manazuru, Kokurakita-ku, Kitakyushu 803-8580, Japan

## Abstract

A spheroid is known as a three-dimensional culture model, which better simulates the physiological conditions of stem cells. This study is aimed at identifying genes specifically expressed in spheroid-cultured human periodontal ligament mesenchymal stem cells (hPDLMSCs) using RNA-seq analysis to evaluate their functions. Transcriptome analysis was performed using spheroid and monolayer cultures of hPDLMSCs from four patients. Cluster and Gene Ontology analyses revealed that genes involved in cell-cell adhesion as well as the G2/M and G1/S transitions of mitotic cell cycles were strongly expressed in the monolayer culture group. However, genes involved in the negative regulation of cell proliferation, histone deacetylation, and bone morphogenetic protein signaling were strongly expressed in the spheroid culture group. We focused on the transcription factor nuclear receptor subfamily 4 group A member 2 (*NR4A2*) among the genes that were strongly expressed in the spheroid culture group and analyzed its function. To confirm the results of the transcriptome analysis, we performed real-time polymerase chain reaction and western blotting analyses. Interestingly, we found that the mRNA and protein expressions of NR4A2 were strongly expressed in the spheroid-cultured hPDLMSCs. Under osteogenic differentiation conditions, we used siRNA to knock down NR4A2 in spheroid-cultured hPDLMSCs to verify its role in osteogenesis. We found that NR4A2 knockdown significantly increased the levels of mRNA expression for osteogenesis-related genes alkaline phosphatase (*ALP*), Osteopontin (*OPN*), and type 1 collagen (*COL1*) (Student's paired *t*-test, *p* < 0.05). ALP activity was also significantly increased when compared to the negative control group (Student's paired *t*-test, *p* < 0.05). Additionally, spheroid-cultured hPDLMSCs transfected with siNR4A2 were cultured for 12 days, resulting in the formation of significantly larger calcified nodules compared to the negative control group (Student's paired *t*-test, *p* < 0.05). On the other hand, NR4A2 knockdown in hPDLMSC spheroid did not affect the levels of chondrogenesis and adipogenesis-related genes under chondrogenic and adipogenic conditions. These results suggest that NR4A2 negatively regulates osteogenesis in the spheroid culture of hPDLMSCs.

## 1. Introduction

Periodontal ligament mesenchymal stem cells (PDLMSCs) are multipotent and can differentiate into bone, cartilage, and adipose tissues [1]. This property has led to their use in regenerative therapy for periodontal tissue that has been lost to periodontitis [2]. Iwata et al. [2–4] reported that cell sheets made from human PDLMSCs (hPDLMSCs) could regenerate periodontal tissue in both dogs and humans. We also previously found that hPDLMSC culture spheroids and hPDLMSC-human umbilical vein endothelial cells (HUVECs) cocultured spheroids enhance the regenerative therapy of periodontal tissue when compared to monolayer-cultured hPDLMSCs [5].

A spheroid is a three-dimensional aggregation of cells and thus is known as a three-dimensional culture model. Spheroid cultures are regarded as more physiologically relevant, as the character of cells is better preserved [6], and the differentiation ability of stem cells is generally enhanced in three-dimensional culture conditions. For example, neuronal differentiation of embryonic stem cells (ESCs) is enhanced in an embryoid body culture when compared to a two-dimensional monolayer cell culture [7]. Another fascinating feature of mesenchymal stem cell (MSC) spheroids is that their formation *in vitro* extends the replicative lifespan of MSCs and delays cellular senescence [6]. Several studies have shown that the expression levels of pluripotency marker genes (*NANOG*, *SOX2*, and *OCT4*) are increased in MSCs [8, 9]. We also found that NANOG and OCT4 were expressed at the mRNA and protein levels in hPDLMSC spheroids; notably, this expression was higher than in the monolayer cultures, which is consistent with previous studies [10]. Recently, we have also shown that hPDLMSC spheroids have superior osteogenic and periodontal tissue regenerative abilities compared to monolayer-cultured hPDLMSCs [6, 10]. Nevertheless, the mechanism of these hPDLMSC spheroid culture properties is not fully understood.

Nuclear receptor subfamily 4 group A member 2 (NR4A2) is an orphan nuclear receptor and transcription factor belonging to the NR4A family. It shows a high degree of similarity to other members of the NR4A family (NR4A1 and NR4A3), especially in the DNA-binding domain [11]. NR4A2 is also known as NURR1, NOT, TINUR, and NGFI-B*β*. NR4A2 expression is rapidly induced in response to various factors, such as fatty acids, prostaglandins, calcium, growth factors, and peptide hormones [12, 13]. Furthermore, NR4A2 has been known to recognize specific DNA sequences, such as NGFI-B response element (NBRE), in a ligand-independent manner to induce downstream gene expression [14]. NR4A2 has also been found to be expressed in the subcellular regions of the brain; NR4A2-deficient mice have significant deficiencies in dopamine-producing neurons in the midbrain and die soon after birth [15]. It has also been shown that genetic mutations in *NR4A2* are found in some cases of familial Parkinson's disease [16]. More recently, it has been reported that NR4A2 is also involved in tumorigenesis [17], as well as NR4A2 expression being upregulated in prostatospheroids [18]. However, the behavior and role of NR4A2 in MSC spheroids are currently unknown.

In this study, we performed genomic analysis (transcriptome analysis) of hPDLMSC spheroid cultures and compared them to the two-dimensional monolayer cultures. This is the first study to perform transcriptome analysis on hPDLMSC spheroids and monolayer cultures. We hypothesize that genes found by transcriptome analysis may be involved in the properties of hPDLMSC spheroids. We focused on NR4A2, which was found to be highly expressed in hPDLMSC spheroid cultures, and analyzed its function in osteogenesis induction.

## 2. Materials and Methods

### 2.1. Cell Culture

hPDLMSCs were obtained from extracted wisdom teeth that were not affected by periodontal disease. These wisdom teeth were extracted at Kyushu Dental University Hospital. After extraction, wisdom teeth were washed five times with phosphate-buffered saline (PBS) (Thermo Fisher Scientific, Waltham, MA, USA) containing antibiotics (100 U/mL penicillin and 100 *μ*g/mL streptomycin; Wako, Osaka, Japan). The periodontal ligament (PDL) was stripped from the middle one-third of the tooth root surface and was digested in a solution of 1 mg/mL type 1 collagenase (Wako) and 1200 pU/mL dispase (Wako) in *α*-MEM (Minimum Essential Medium) (Thermo Fisher Scientific) for 1 h at 37°C while shaking. Single-cell suspensions were obtained by passing the cells through a 70 *μ*m strainer (Corning, NY, USA). Cells were cultured in 100 mm culture dishes (Iwaki, Shizuoka, Japan) with *α*-MEM containing 10% fetal bovine serum (FBS; Biocera, Nuaillé, France), 100 U/mL penicillin, and 100 *μ*g/mL streptomycin in a humidified atmosphere with 5% CO_2_ at 37°C. After 12 h, new medium was added to remove unattached cells. Subcultures were performed every 4–5 d until passage 5. To confirm the differentiation potency of the cells, osteogenesis, chondrogenesis, and adipogenesis assays were performed (data not shown). In addition, flow cytometry analysis confirmed these cells expressed CD29, 44, 73, 90, 105, and 106 and did not express CD34 and 45 (data not shown). hPDLMSCs were obtained with the approval of the Kyushu Dental University Ethics Committee (Protocol # 14–21). All patients who donated teeth to harvest the PDL gave their consent for the use of the teeth in this study. hPDLMSCs from four patients ((1) 28 from 25-year-old male, (2) 18 from 26-year-old male, (3) 18 from 30-year-old male, and (4) 28 from 25-year-old female) were used for the transcriptome analysis and NR4A2 functional analysis under osteogenic, chondrogenic, and adipogenic conditions.

### 2.2. Spheroid Formation

hPDLMSC spheroids were formed with microwell chips, which were prepared as previously described [10, 19, 20]. In this study, the microwell chip with 500 *μ*m diameter and 500 *μ*m depth was designed. The microwell structures of the chip were fabricated by a milling system (PMT Corp., Fukuoka, Japan). The surface of microwell chips was coated with a layer of platinum by using an ion sputter unit (Hitachi High-Tech Science Systems Corp., Ibaraki, Japan). The chips were immersed in 5 mM polyethylene glycol in ethanol solution and washed with ultrapure water, followed by rinsing 50% ethanol for removal of the unattached polyethylene glycol and sterilization. The microwell chips were immersed in medium for cell culture before use. hPDLMSCs were cultured with 2.000 cells per well in the microwell chips in *α*-MEM medium containing 10% FBS. The medium was changed every other day.

### 2.3. Transcriptome Analysis

Four types of hPDLMSCs were collected from the extracted wisdom teeth of four patients, and these were cultured in microwell chips for 3 days to prepare the spheroids. hPDLMSCs from the same four patients cultured in a monolayer for 3 days were prepared as control groups. RNA was collected from both groups using an RNeasy mini kit (Thermo Fisher Scientific), according to the manufacturer's instructions. RNA-seq was performed using the Illumina HiSeq system (San Diego, CA, USA). Adaptor sequences and low-quality bases of fastq files were trimmed by Trimmomatic (v. 0.35). Sequencing reads were aligned to the human reference genome (hg38) using the HISAT2 (v. 2.1.0) software. Cufflinks (v. 2.2.1) was used to normalize uniquely mapped reads as fragments per kilobase of exon per million reads mapped (FPKM). To detect differentially expressed genes (DEGs) between the two groups, we used Cuffcompare, Cuffquant, and Cuffdiff tools that calculate a fold change for every gene, *p* value, and false discovery rate (FDR) using the Benjamini-Hochberg correction (*q* value) in the Cufflinks packages. DEGs were defined when the *q* value < 0.05, and the fold change of FPKM was ≥2.0. The hg38 reference and the Refseq annotation were obtained from the UCSC Genome Browser (https://genome.ucsc.edu/). Enriched Gene Ontology (GO) terms were analyzed using the Database for Annotation, Visualization, and Integrated Discovery (DAVID; https://david.ncifcrf.gov/) with the annotation dataset of GO biological processes. Specific GO terms were obtained from the NCBI database (ftp://ftp.ncbi.nlm.nih.gov/gene/DATA/). Most of the plots and graphs of RNA-seq analysis were visualized using ggplot2 (v. 3.0.0) and other R packages. Clustering analysis was performed using the function hclust of R packages.

### 2.4. Quantitative Real-Time Polymerase Chain Reaction Assay

To validate the changes in mRNA expression, quantitative real-time polymerase chain reaction (qRT-PCR) was performed using a StepOne™ real-time system (Thermo Fisher Scientific) as previously described [21]. Total RNA was extracted from monolayer and spheroid-cultured hPDLMSCs using an RNeasy mini kit (Qiagen, Hilden, Germany), according to the manufacturer's instructions. The reverse transcription reaction was performed with a High-Capacity cDNA Reverse Transcription kit (Thermo Fisher Scientific) at 37°C for 60 min and 95°C for 5 min. PCR products were detected using FAST SYBR® Green Master Mix (Thermo Fisher Scientific) with the respective primer sequences shown in Table 1 Relative changes in gene expression were calculated using the comparative CT method. Total cDNA abundance between samples was normalized using primers specific to the *GAPDH* gene.

### 2.5. RNA Interference

Small interfering RNAs (siRNAs) for NR4A2 (s9787) and the nontargeting control (4390843) were purchased from Thermo Fisher Scientific. Forty thousand hPDLMSCs were plated into six-well plates and microwell chips for the monolayer group and the spheroid groups, respectively, and cultured for 3 days. Each siRNA was used to transfect the hPDLMSC culture via the Lipofectamine RNAiMAX reagent (Thermo Fisher Scientific). These cells were then cultured for experiments and harvested for RNA and whole-cell lysate preparation.

### 2.6. Alkaline Phosphatase Activity (ALP) Assay

ALP activity in the spheroid hPDLMSCs was measured using the LabAssay™ ALP system (Wako), following the manufacturer's protocols. hPDLMSC spheroids or monolayer-cultured hPDLMSCs treated with siNR4A2 and the nontargeting control or NR4A2 overexpression vector and control vector were cultured in hMSC Osteogenic Differentiation Medium (Lonza, Basel, Switzerland) as an osteoinductive medium (OIM) for 7 days. Cells were washed with PBS and lysed using cell lysis buffer (Wako). Samples were added to *p*-nitrophenyl phosphate and incubated at 37°C for 15 min; then, the reaction stopping solution was added, and the optical density of *p*-nitrophenol was measured at the absorbance at 405 nm using a microplate reader (Molecular Devices LLC, Union City, CA, USA).

### 2.7. Western Blotting Analysis

Western blotting was performed as described previously [21]. Cells from monolayer and spheroid cultures were rinsed with cooled PBS, and whole-cell lysates were prepared by adding Cell Lysis Buffer M (Wako) supplemented with sodium orthovanadate (V) (Wako), phenylmethylsulfonyl fluoride (Nacalai Tesque, Kyoto, Japan), and Halt™ Protease Inhibitor Cocktail (Thermo Fisher Scientific). Protein concentration was measured using a DC™ protein assay kit (Bio-Rad Laboratories, Hercules, CA, USA). Equivalent amounts of the protein were separated using sodium dodecyl sulfate-polyacrylamide gel electrophoresis (SDS-PAGE) and transferred to an Immun-Blot PVDF Membrane (Bio-Rad). Nonspecific binding sites were blocked by immersing the membrane in Blocking One buffer (Nacalai Tesque) for 30 min at 20°C. Membranes were incubated overnight with diluted primary antibodies for NR4A2 (PA5-13416; Carlsbad, Invitrogen, CA, USA), 1 : 1000 and GAPDH (G8795; Sigma-Aldrich, St Louis, MO, USA), 1 : 20,000 at 4°C. Membranes were then reacted with KPL Antibodies and Conjugates Anti-Rabbit IgG (H+L) Antibody, Human Serum Adsorbed, and Peroxidase-Labeled (Seracare, Milford, MA, USA) secondary antibodies for 1 h at 20°C. After washing the membrane, chemiluminescence was detected using ImmunoStar LD (Wako) and ChemiDoc™ XRS Plus system (Bio-Rad).

### 2.8. Nodule Formation Assay

hPDLMSC spheroids were formed after a 3-day culture on microwell chips. After transfection of the siNR4A2 or nontargeting control, the culture medium was changed to hMSC Osteogenic Differentiation Medium (Lonza) for 12 days to induce mineralization. Cells were fixed with 99.5% methyl alcohol (Wako) for 10 min and stained with a calcified nodule staining kit (Cosmo Bio, Tokyo, Japan) according to the manufacturer's instructions. To quantify mineralization, the alizarin red-colored area inside the hPDLMSC spheroid and area of hPDLMSC spheroid were measured using ImageJ (National Institutes of Health, Bethesda, MD, USA). The positivity rate for the alizarin red hPDLMSC spheroid stain was calculated using calculation, alizarin red-colored area inside the hPDLMSC spheroid/area of hPDLMSC spheroid.

### 2.9. Cell Proliferation Assay

hPDLMSC spheroids were formed after a 3-day culture on microwell chips. After transfection of the siNR4A2 or nontargeting control, these spheroids were cultured for 3 and 7 days. Viability was assessed using the CellTiter 96 Aqueous One solution cell proliferation assay kit (Promega Corp.) according to the manufacturer's instructions. CellTiter96® Aqueous One Solution was added (20 *μ*L/well) and incubated for another 4 h at 37°C and 5% CO_2_; then, 25 *μ*L/well of 10% SDS was added. The plate was then read at 490 nm.

### 2.10. Overexpression Assay

The nucleotide sequence encoding human NR4A2 (accession no. NM_006186) was amplified from the plasmid Flexi ORF Clone FXC21198 (Kazusa DNA Res. Inst., Chiba, Japan) by standard PCR technique using PrimeSTAR Max DNA polymerase (TaKaRa, Ohtsu, Japan). This nucleotide sequence was inserted into a pcDNA3.1/V5-His vector (Invitrogen). hPDLMSCs were transfected with human NR4A2 vector or control vector by using Lipofectamine 2000 (Invitrogen) according to manufacturer instructions. These cells were then cultured for experiments and harvested for RNA and whole-cell lysate preparation.

### 2.11. Chondrogenesis and Adipogenesis Assay

hPDLMSC spheroids were formed after a 3-day culture on microwell chips. After transfection of the siNR4A2 or nontargeting control, twenty spheroids (40,000 cells) were transferred to a 15 mL tube and cultured with hMSC Chondrogenic Differentiation Medium (Lonza) to induce chondrogenesis. After 7-day culture, the samples were collected to measure the levels of type 2 collagen (*COL2*) and type 10 collagen (*COL10*) expression, chondrogenic markers by real-time PCR. On the other hand, for adipogenesis, twenty spheroids (40.000 cells) were transferred to a 48-well plate and cultured with hMSC Adipogenic Differentiation Medium (Lonza). After 7-day culture, the samples were collected to measure the levels of CCAAT/enhancer-binding protein *α* (*CEBPα*) and peroxisome proliferator-activated receptor *γ* (PPAR*γ*) expression, adipogenic markers by real-time PCR.

### 2.12. Statistical Analysis

The statistics of the NR4A2 functional analysis experiment are presented as the mean ± standard deviation of twelve samples of cells collected from four patients. All experiments were performed at least thrice. Statistical analysis was performed using Excel software (Microsoft, Redmond, WA, USA), using paired Student's *t*-test for comparison; *p* values < 0.05 were considered statistically significant. Power analysis confirmed that the statistical power was greater than 0.8 for all statistical tests in this study. The statistics of RNA-seq are described in detail in Transcriptome Analysis.

## 3. Results

### 3.1. Gene Profile for Spheroid-Cultured hPDLMSCs by RNA-seq

**Figure 1:**
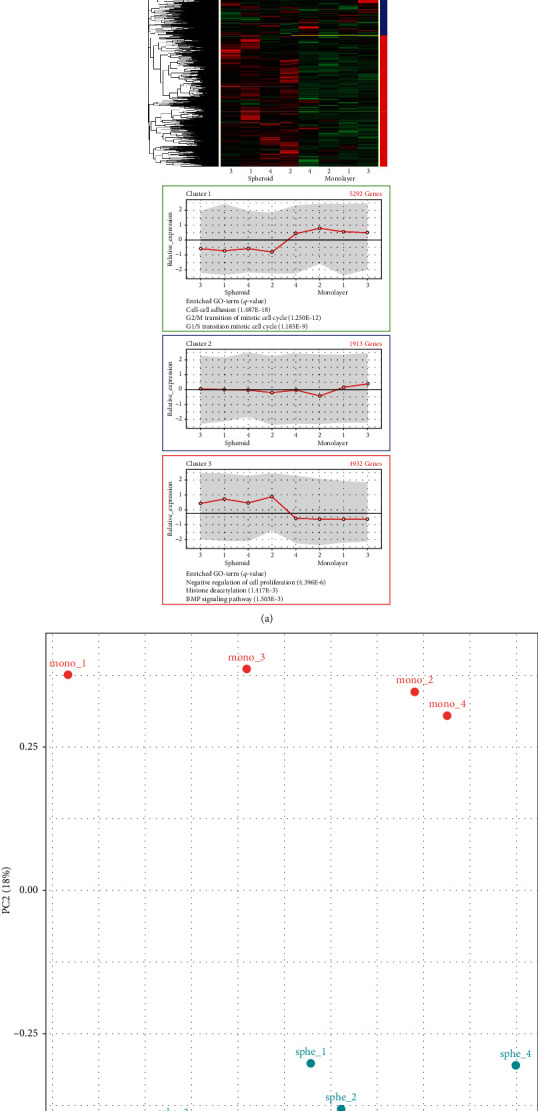
Gene expression profile in each individual sample analyzed by RNA-sequencing. RNA-seq detected 12,137 genes (FPKM ≥ 1.0), which were categorized into 3 patterns by hierarchical clustering approach (a; left). Cluster 1, which consists of genes upregulated in monolayer culture, and Cluster 3, which consists of genes upregulated in spheroid culture, were analyzed using DAVID to determine specific enriched GO terms. The figure shows the representative significantly enriched GO terms (Benjamini-Hochberg < 0.05) (a; right). Principal component analysis (PCA) was performed using 12,137-gene (FPKM ≥ 1.0) expression datasets. The PCA plot shows two clearly distinguishable cell culture samples depicted as red (=monolayer) and blue (=spheroid) clusters (b).

The sequenced reads were aligned with the human reference genome (hg38) using HISAT2, and then, the gene expression levels of each sample were quantified as in FPKM using Cufflinks packages. To remove the low-expression genes between the two groups, only the RefSeq genes expressed in at least one of the groups with FPKM ≥ 1 were used for this study, which consisted of 12,137 genes. These genes were divided into three different gene clusters exhibiting distinct expression patterns (Figure 1(a)). Cluster 1 comprised of 5,292 genes that were upregulated in the monolayer culture when compared with the spheroid culture; conversely, Cluster 3 comprised of 4,932 genes, which were upregulated in the spheroid culture when compared with the monolayer culture. Cluster 2 comprised 1,913 genes, which were unaffected by culture conditions. The genes in each cluster were markedly enriched by specific GO terms. Genes in Cluster 1 possessed specific GO terms, such as cell-cell adhesion, G2/M transition of mitotic cell cycle, and G1/S transition of mitotic cell cycle. On the other hand, genes in Cluster 3 possessed specific GO terms, such as negative regulation of cell proliferation, histone deacetylation, and bone morphogenetic protein (BMP) signaling (Figure 1(a)). The GO term analysis suggests that spheroid culture systems can induce epigenetic changes, such as histone deacetylation, which may cause cell cycle arrest. Moreover, our RNA-sequencing analysis showed that the whole gene expression profile of the spheroid culture was dramatically different from the monolayer culture profile (Figure 1(b)).

**Figure 2:**
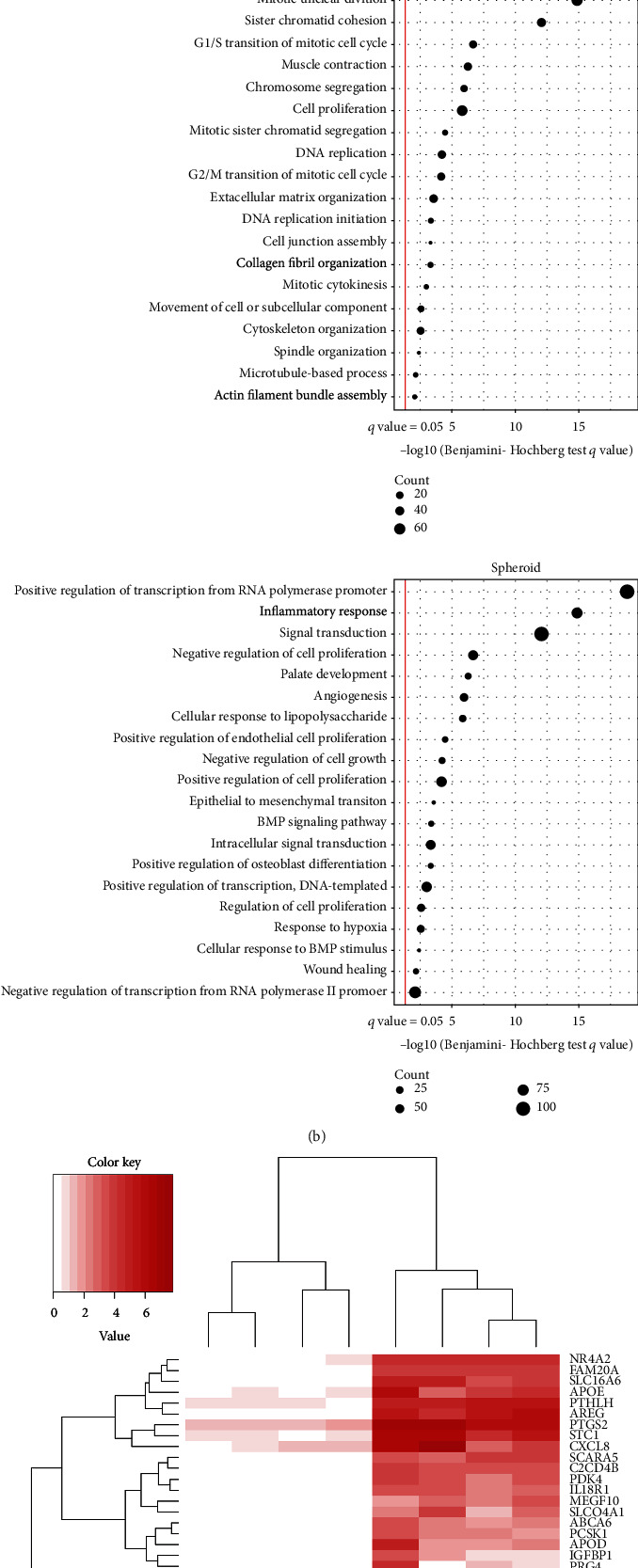
Statistical analysis revealed 2,196 (1,061 upregulated and 1,135 downregulated) DEGs between monolayer and spheroid, using the criteria of *q* value < 0.05 and fold change ≥ 2.0. The red and gray plots represent DEGs and non-DEGs, respectively (a). Top 20 enriched GO terms in the upregulated DEGs in monolayer culture or spheroid culture were determined by DAVID. Left side of the red line designates Benjamini-Hochberg < 0.05 (b). Heatmap shows expression level (on a loge scale) of top 20 upregulated DEGs in monolayer culture or spheroid culture (c). The bar plot shows fold change (on a Log_2_ scale) between the spheroid and monolayer culture for stem cell maintenance population genes. The red and gray bars represent DEGs and non-DEGs, respectively (d).

To detect the potential key regulators associated with spheroid culture, we analyzed the DEGs (fold change ≥ 2, *q* value (FDR with Benjamini‐Hochberg correction) < 0.05)) between the spheroid and monolayer cultures, successfully identifying 2,196 genes of them (Figure 2(a)). To investigate differences between the spheroid and monolayer cultures, GO term enrichment analysis was performed using the upregulated DEGs in both the monolayer and spheroid cultures. The top 20 significantly enriched GO biological process terms (*q* value < 0.05) were identified (Figure 2(b)). Most of the top 20 GO terms that were identified from upregulated DEGs in the monolayer cultures were involved in the cell cycle. Interestingly, upregulated DEGs in the spheroid cultures possessed GO terms related to osteoblastic differentiation, such as positive regulation of osteoblast differentiation and BMP signaling pathway.  Figure 2(c) shows that the expression levels of the top 20 up- or downregulated DEGs were observed for each individual sample. As a previous study suggested that spheroid cultures enhance the stemness of hPDLMSCs [10], we focused on stemness-related genes.  Figure 2(d) shows that most of the genes that were categorized under the GO term stem cell population maintenance were upregulated in spheroid cultures. Among the DEGs between the spheroid and monolayer culture groups, we extracted genes with Log_2_(fold change) ≥ 6, *q* value < 0.05, and FPKM > 50 in the spheroid culture group (Table 2). Transcription factors regulate the expression of various genes and may be involved in the diverse functions of stem cell spheroids. We then focused on the transcription factor NR4A2 for functional analysis to search for factors that could be master genes in stem cell differentiation, such as Runx2 and osterix in osteoblast differentiation and NFATc1 in osteoclast differentiation.

### 3.2. NR4A2 Expression in hPDLMSC Spheroids

**Figure 3:**
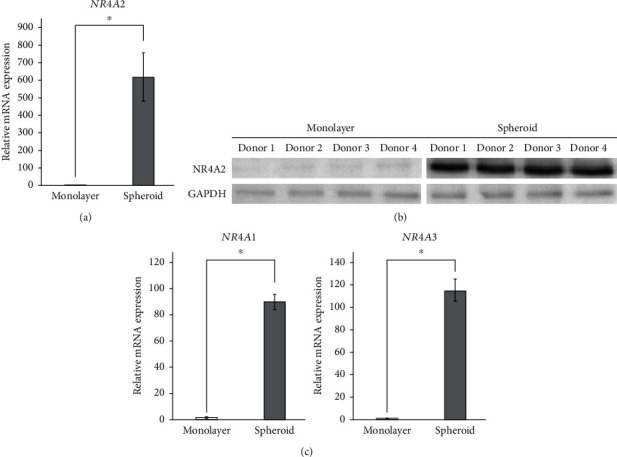
Validation of NR4A2 expression in spheroid cultures of hPDLMSC. (a) Expression of NR4A2 mRNA in monolayer and spheroid-cultured hPDLMSC on day 3 by real-time RT-PCR. ^∗^*p* < 0.05 (compared with monolayer culture). (b) Expression of NR4A2 protein in monolayer and spheroid-cultured hPDLMSC on day 3 by western blotting. (c) NR4A1 and NR4A3 mRNA expression in monolayer and spheroid-cultured hPDLMSC on day 3 by real-time RT-PCR. ^∗^*p* < 0.05 (compared with monolayer culture). All experiments were performed with samples from four donors.

*NR4A2* mRNA expression in hPDLMSC spheroids was confirmed by real-time PCR analysis. The levels of *NR4A2* mRNA expression in spheroid-cultured hPDLMSCs were approximately 100 times higher than that in monolayer-cultured hPDLMSCs, consistent with the results of the transcriptome analysis (Figure 3(a)). We also performed western blotting analysis to confirm the protein expression of NR4A2. NR4A2 protein expression was not found in monolayer cultures of hPDLMSCs; however, it could be clearly identified in the spheroid cultures of hPDLMSC (Figure 3(b)). We also examined NR4A1 and NR4A3 expression because they belong to the same family as NR4A2. Similar to *NR4A2*, the mRNA expression levels of *NR4A1* and *NR4A3* were much higher in hPDLMSC spheroids than in monolayer-cultured hPDLMSCs (Figure 3(c)).

### 3.3. Role of NR4A2 in hPDLMSC Spheroids during Osteogenesis

**Figure 4:**
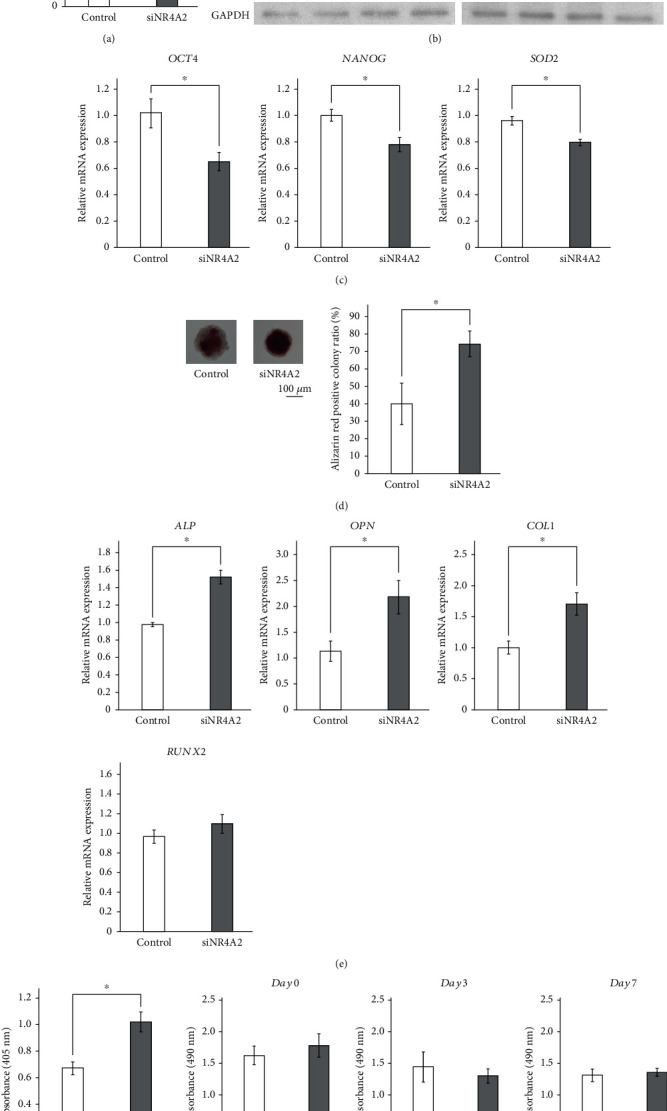
Enhancement of osteogenesis in hPDLMSC spheroid treated with NR4A2 siRNA through ALP activation. (a, b) Confirmation of silencing of NR4A2 by siRNA in spheroid-cultured hPDLMSC. NR4A2 siRNA decreased mRNA expression of NR4A2 to about 30%. Expression of NR4A2 also was reduced by siNR4A2. (c) Effects of siNR4A2 on *OCT4*, *NANOG*, and *SOD2* mRNA expression in spheroid hPDLMSCs. The expression of *OCT4*, *NANOG*, and *SOD2* mRNA was examined by real-time RT-PCR. ^∗^*p* < 0.05 (compared with control). (d) Nodule formation of hPDLMSC spheroid treated with siNR4A2 or control in OIM condition. Cells were transfected with siNR4A2 or control and then cultured for 12 days in osteoinductive medium and stained with alizarin red. (e) Effects of siNR4A2 on ALP, OPN, Col1, and RUNX2 mRNA expression in spheroid hPDLMSCs cultured with OIM. On day 7, the expression of ALP, OPN, Col1, and RUNX2 mRNA was examined by real-time RT-PCR. ^∗^*p* < 0.05 (compared with control). (f) Effects of siNR4A2 on ALP activity in spheroid hPDLMSCs cultured with OIM. On day 7, ALP activity was measured. ^∗^*p* < 0.05 (compared with control). (g) Effect of siNR4A2 on proliferation in spheroid hPDLMSCs on days 0, 3, and 7. All experiments were performed with samples from four donors.

To examine the function of NR4A2 in hPDLMSC spheroids, we performed an siRNA knockdown assay. First, we checked the knockdown efficacy of siRNA. The levels of NR4A2 mRNA expression in hPDLMSCs treated with siNR4A2 were decreased by approximately 70% when compared to that in the negative control siRNA (Figure 4(a)). Western blotting analysis revealed that NR4A2 protein expression in siNR4A2-treated hPDLMSC spheroids was also reduced when compared with that in the control (Figure 4(b)). We also examined the effect of siNR4A2 on the mRNA expression of *NANOG* and *OCT4*, which are stemness markers, and *SOD2*, an oxidative stress marker, in spheroid-cultured hPDLMSCs. NR4A2 knockdown significantly decreased the mRNA levels of *NANOG*, *OCT4*, and *SOD2* compared to that in the negative control (Figure 4(c)). These data suggested that NR4A2 knockdown reduced stemness in spheroid-cultured hPDLMSCs.

Next, we performed a nodule formation assay with hPDLMSC spheroids to clarify the novel role of NR4A2 in osteogenesis. We cultured spheroids of hPDLMSCs treated with nontargeting control siRNA or siNR4A2 in OIM for 12 days. Under osteogenic conditions, hPDLMSCs treated with both siRNA vectors formed alizarin red-positive calcium deposits. The areas of the alizarin red-positive nodules from spheroid-derived hPDLMSCs with siNR4A2 were significantly greater than that of the negative control siRNA-treated cells (Figure 4(d)).

To investigate whether osteogenic genes were induced in spheroid hPDLMSCs treated with siNR4A2 in OIM conditions, the expression of *RUNX2*, *ALP*, *OPN*, and *COL1* mRNA was measured by real-time PCR. The expression of *ALP*, *OPN*, and *COL1* mRNA in spheroid-cultured hPDLMSCs targeted with siNR4A2 was significantly increased after 7 days of culture when compared with that in the negative control. However, the mRNA expression of *RUNX2*, at an early stage of osteoblast differentiation, was comparable to that in the negative control (Figure 4(e)). We also examined the effect of siNR4A2-treated hPDLMSCs in OIM on the ALP activity. The ALP activity of hPDLMSCs in spheroid cultures treated with siNR4A2 was significantly increased compared with that in the negative control vector-treated spheroid culture (Figure 4(f)). On the other hand, siNR4A2 had no effect on hPDLMSC proliferation (Figure 4(h)). These data suggest that NR4A2 negatively regulates osteogenesis of hPDLMSCs in spheroid cultures.

**Figure 5:**
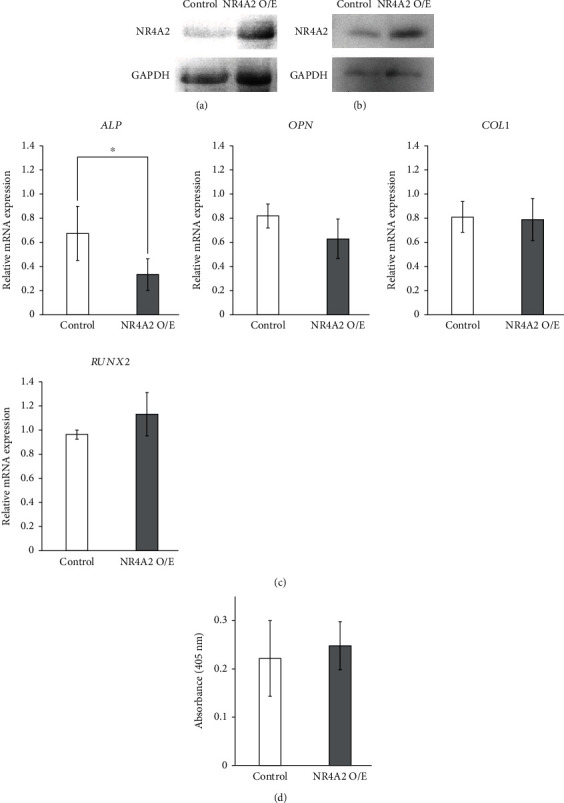
Effects of NR4A2 overexpression on osteogenesis in hPDLMSC spheroids. (a, b) Confirmation of overexpression of NR4A2 in spheroid-cultured hPDLMSC. Transfection of NR4A2 overexpression vector increased NR4A2 protein expression in monolayer and spheroid-cultured hPDLMSCs. (c) Effects of NR4A2 overexpression on ALP, OPN, Col1, and RUNX2 mRNA expression in spheroid hPDLMSCs cultured with OIM. On day 7, the expression of ALP, OPN, Col1, and RUNX2 mRNA was examined by real-time RT-PCR. ^∗^*p* < 0.05 (compared with control). (d) Effects of NR4A2 overexpression on ALP activity in spheroid-cultured hPDLMSCs cultured with OIM. On day 7, ALP activity was measured. ^∗^*p* < 0.05 (compared with control). All experiments were performed with samples from four donors.

To further elucidate the role of NR4A2 in hPDLMSC spheroids during osteogenesis, we performed NR4A2 overexpression assays. First, we checked the effect of the NR4A2 overexpression vector. Western blotting analysis showed that NR4A2 protein expression in monolayer-cultured hPDLMSCs treated with NR4A2 overexpression vector was increased when compared to that in the control vector (Figure 5(a)). After transfection NR4A2 overexpression or control vectors, hPDLMSCs were cultured in microwell chips for spheroid formation in OIM for 7 days. NR4A2 protein expression in hPDLMSC spheroids treated with NR4A2 overexpression vector was comparable to those treated with control vector (Figure 5(b)). The real-time PCR assay revealed that the expression of *Runx2*, *OPN*, and *COL1* mRNA in spheroid-cultured hPDLMSCs treated with NR4A2 overexpression vector was comparable to after 7 days of culture when compared with that in the negative control (Figure 5(c)). Furthermore, the ALP activity of hPDLMSCs in spheroid cultures treated with NR4A2 overexpression vector was also comparable to that in the negative control vector-treated spheroid culture (Figure 5(d)).

### 3.4. Role of NR4A2 in hPDLMSC Spheroids during Adipogenesis and Chondrogenesis and in Monolayer-Cultured hPDLMSC during Osteogenesis

**Figure 6:**
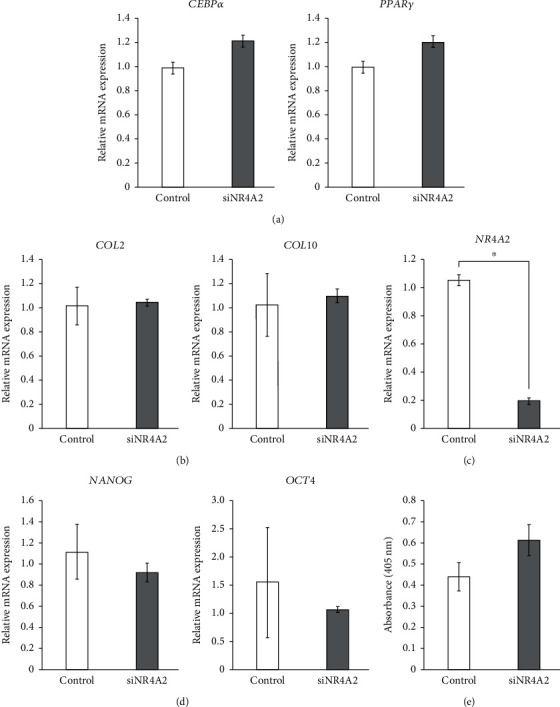
Effects of NR4A2 on chondrogenesis and adipogenesis in hPDLMSC spheroids and osteogenesis in monolayer-cultured hPDLMSCs. (a) Effects of siNR4A2 on CEBP*α* and PPAR*γ* mRNA expression in monolayer-cultured hPDLMSCs with adipogenic differentiation medium. On day 7, the expression of CEBP*α* and PPAR*γ* mRNA was examined by real-time RT-PCR. (b) Effects of siNR4A2 on COL2 and COL10 mRNA expression in monolayer- cultured hPDLMSCs with chondrogenic differentiation medium. On day 7, the expression of COL2 and COiL10 mRNA was examined by real time RT-PCR. (c) Confirmation of silencing of NR4A2 by siRNA in monolayer-cultured hPDLMSC. NR4A2 siRNA decreased mRNA expression of NR4A2. (d) Effects of siNR4A2 on Nanog and Oct4 mRNA expression in monolayer-cultured hPDLMSCs. The expression of NR4A1 and NR4A3 mRNA was examined by real-time RT-PCR. (e) Effects of siNR4A2 on ALP activity in monolayer-cultured hPDLMSCs cultured with OIM. On day 7, ALP activity was measured. All experiments were performed with samples from four donors.

To examine the role of NR4A2 in hPDLMSC spheroids on adipogenesis and chondrogenesis, siNR4A2 and control siRNA-treated hPDLMSC spheroids were cultured under adipogenic and chondrogenic conditions for 7 days. The mRNA expression of *CEBPα* and *PPARγ*, as markers of adipogenesis, and the mRNA expression of *COL2* and *COL10*, as markers of chondrogenesis, were measured by real-time PCR. The expression of *CEBPα* and *PPARγ* mR*ΝΑ* in spheroid-cultured hPDLMSCs targeted with siNR4A2 was comparable to negative control after 7 days of adipogenic culture (Figure 6(a)). Similar to adipogenesis, the levels of *COL2* and *COL10* mRNA expression in siNR4A2-treated hPDLMSC spheroid were also comparable to control siRNA (Figure 6(b)). These data suggest that NR4A2 does not affect adipogenesis and chondrogenesis of hPDLMSCs in spheroid culture.

Finally, to clarify the function of NR4A2 in monolayer culture hPDLMSCs, we performed an siRNA knockdown assay. The levels of NR4A2 mRNA expression in monolayer-cultured hPDLMSCs treated with siNR4A2 were decreased compared to that in the negative control siRNA (Figure 6(c)). Unlike spheroid culture, NR4A2 knockdown did not affect Nanog and Oct4 mRNA expression in monolayer-cultured hPDLMSCs (Figure 6(d)). We also examined the effect of siNR4A2-treated hPDLMSCs of monolayer culture in OIM on the ALP activity. The ALP activity in monolayer-cultured hPDLMSCs treated with siNR4A2 was comparable to the negative control (Figure 6(e)). These data suggested that the effect of NR4A2 is specific for hPDLMSC spheroid under osteogenic conditions.

## 4. Discussion

We performed transcriptomic analysis to identify DEGs between the spheroid and monolayer-cultured hPDLMSCs using next-generation sequencing. We identified *NR4A2*, among other genes that are highly expressed in hPDLMSCs. Frith et al. [22] also performed microarray analysis using bone marrow-derived MSCs that were cultured in spheroid and monolayer cultures and found the top 50 upregulated genes in each type of culture. Some of the 50 genes upregulated in the bone marrow-derived MSC spheroid culture group included *SLC16A6*, *AREG*, *NR4A2*, and *PTHLH*, which are also shown in Table 1. However, *STC1*, *CXCL8*, *PTGS2*, and *FAM20A* were not among them. Thirty-five of the top 50 genes were also significantly elevated in our analysis when compared to that in the monolayer culture group. Similarly, in the monolayer culture group, 36 of the top 50 genes that were upregulated compared to that in the spheroid culture group were also significantly upregulated in our analysis [22]. These data suggest that the gene profiles in spheroids with different MSCs are somewhat consistent. In our previous transcriptome analysis of bone marrow-derived MSCs and PDL-derived MSCs, the expression of the *HOX* gene differed between the two MSCs [23]. In other words, MSCs derived from different anatomical sites have marked differences in gene expression and positional memory, although they meet the same general characterization criteria. This may be the reason why our results were not completely consistent with those of Frith et al. [22].

Although there were several DEGs obtained between the monolayer and spheroid-cultured hPDLMSCs, we focused on NR4A2, a versatile orphan receptor, which is strongly expressed in hPDLMSC spheroids. The NR4A family is known to have versatile functions in cellular homeostasis [24]. NR4A2 knockdown significantly increased calcification of spheroid-cultured hPDLMSCs (Figure 4(d)). Our data suggests that NR4A2 negatively regulates osteoblastic differentiation in spheroid-cultured hPDLMSCs. However, a few studies show the opposite effect of NR4A2 in osteoblast differentiation. The first shows that the activity of ALP was decreased in NR4A2 siRNA-treated MC3T3-E1 cells, a mouse osteoblast cell line [25]. The second shows that NR4A2 transactivates the osteocalcin promoter and induces osteocalcin mRNA expression [26]. These reports suggest that NR4A2 promotes osteoblast differentiation. However, this opposite effect may be due to the differences between MSCs and osteoblasts. Regarding our hypothesis, NR4A2-silenced cultures of MSCs harvested from the dental pulp significantly enhanced mineralization when compared to controls [27]. Further studies using stem cells derived from other tissues, such as bone marrow-derived MSCs, are needed to fully elucidate the role of NR4A2 in the differentiation of MSCs. hPDLMSCs are well known to differentiate into osteoblasts, cementoblasts, and fibroblasts [1], whereas after NR4A2 knockdown, the expression of *NANOG* and *OCT4* was partially reduced (Figure 4(c)). This result suggests a loss of stemness and increased bone differentiation under osteogenic conditions. However, the genes involved in the multilineage differentiation ability of hPDLMSCs and in the osteoblastic differentiation process currently remain unclear; thus, further investigation is required because osteogenesis is influenced by many factors. Although NR4A2 knockdown significantly increased calcification of spheroid-cultured hPDLMSCs, NR4A2 overexpression in hPDLMSCs spheroid did not decrease the mRNA levels of osteogenesis-related genes and ALP activity unexpectedly. The levels of NR4A2 mRNA and protein expression in spheroid-cultured hPDLMSC are originally much higher than in monolayer-cultured hPDLMSCs, and overexpression of NR4A2 may not affect osteogenesis because hPDLMSC spheroid expresses and produces sufficient NR4A2.

In this study, transcriptomic analysis revealed that NR4A2 was more strongly expressed in hPDLMSCs cultured in spheroids than in monolayers. Moreover, we discovered via real-time PCR that not only NR4A2 but also NR4A1 and NR4A3 were intensely upregulated in hPDLMSCs cultured in spheroids. Knockdown of NR4A2 could only partially inhibit the expression of *NANOG* and *OCT4*, stemness markers in spheroid-cultured hPDLMSCs, which might be because it was compensated by the expression of NR4A1 and NR4A3 belonging to the same NR4A family. In the future, we plan to investigate the effect of NR4A2, NR4A1, and NR4A3 on stemness of spheroid-cultured hPDLMSCs. We performed an *in vitro* only study; thus, the physiological significance of NR4A2 is not clear. In vivo analysis using knockout mice is necessary to clarify the physiological significance of NR4A2. However, it is known that NR4A2-deficient mice show a marked deficiency of dopamine-producing neurons in the midbrain and die soon after birth [15]. Because NR4A2-deficient mice are extremely difficult to maintain long-term postnatally, there are few analyses of dysfunctions in adults. The phenotype of NR4A2-deficient mice in bone and periodontal tissue is unknown. To clarify the role of NR4A2 in MSCs, analysis using conditionally deficient mice is necessary.

The NR4A2 protein forms a complex with several association molecules and induces the expression of target genes by recognizing specific sequences, such as the nuclear receptor binding element (NRBE), known by its sequence of **A**AAGGTCA, and the Nur response element (NurRE) [28–32]. NR4A2 knockdown enhanced OIM-induced osteogenesis in spheroid-cultured hPDLMSCs mediated by the upregulation of *ALP* and *COL1* (Figure 4). We searched the promoter/enhancer region of ALP and type 1 collagen by database analysis. We found several candidate binding sites for NR4A2 in their promoter and enhancer regions. This suggests that NR4A2 may directly regulate *ALP* and *COL1* gene expression. However, NR4A2 is known to form a heterodimer with the retinoic X receptor [24]. Taken together, we suggest that NR4A2 may regulate *ALP* gene expression in a nondirect manner by forming a complex with the retinoic acid receptor. In the future, we plan to conduct mutation assays using the *ALP* promoter to elucidate this mechanism in detail.

We have identified NR4A2, an orphan receptor and transcription factor, as a protein that is highly expressed in spheroid-cultured hPDLMSCs by transcriptome analysis. This is the first study to show that NR4A2 negatively regulates osteogenesis in spheroid-cultured hPDLMSCs. To further validate the physiological significance of the hPDLMSC spheroid, we plan to conduct functional analysis studies of the other genes identified in this study.

## 5. Conclusions

RNA-seq analysis revealed that the gene expression profiles of hPDLMSCs in spheroid and monolayer culture groups were significantly different. Cluster analysis and GO term analysis revealed that genes involved in cell-cell adhesion and the G2/M and G1/S transitions of mitotic cell cycle were strongly expressed in the monolayer culture group, whereas genes involved in the negative regulation of cell proliferation, histone deacetylation, and BMP signaling were strongly expressed in the spheroid culture group. Functional analysis of NR4A2, which was highly expressed in the spheroid culture group, suggested that NR4A2 negatively regulated the osteogenesis of spheroid-cultured hPDLMSCs.

## Figures and Tables

**Table 1:** Primer sequences for real-time RT-PCR.

Genes	Gene ID	Sequences of primers	PCR product
*GAPDH*	*2597*	F: 5′-GAAGGTGAAGGTCGGAGTC-′	206
		R: 3′-GAAGATGGTGATGGGATTTC-5′	
*ALP*	*249*	F: 5′-ACGTGGCTAAGAATGTCATC-3′	457
		R: 3′-CTGGTAGGCGATGTCCTTA-5′	
*OPN*	*6696*	F: 5′-CCAAGTAAGTCCAACGAAAG-3′	329
		R: 3′-GGTGATGTCCTCGTCTGTA-5′	
*COL1*	*1277*	F: 5′-AGGGCTCCAACGAGATCGAGATCCG-3′	196
		R: 3′-TACAGGAAGCAGACAGGGCCAACGTCG-5′	
*RUNX2*	*860*	F: 5′-AACCCTTAATTTGCACTGGGTCA-3′	120
		R: 3′-CAAATTCCAGCAATGTTTGTGCTAC-5′	
*NR4A1*	*3164*	F: 5′-AAGCCACATTGTTGCCAAGACCTG-3′	122
		R: 3′-TGGTGTCCCATATTGGGCTTGGAT-5′	
*NR4A2*	*4929*	F: 5′-GTCTCAGCTGCTCGACACG-3′	139
		R: 3′-TTTTGCACTGTGCGCTTAAA-5′	
*NR4A3*	*8013*	F: 5′-TACACCAAGCTGACCATGGACCTT-3′	110
		R: 3′-ATTTGGTACACGCAGGAAGGCTTG-5′	
*OCT4*	*5460*	F: 5′-AGCAAAACCCGGAGGAGT-3′	93
		R: 3′-CCACATCGGCCTGTGTATATC-5′	
*NANOG*	*79923*	F: 5′-TGAACCTCAGCTACAAACAG-3′	134
		R: 3′-TGGTGGTAGGAAGAGTAAAG-5′	
*SOD2*	*6648*	F: 5′-TTCTGGACAAACCTCAGCCC-3′	49
		R: 3′-CGTTTGATGGCTTCCAGCA-5′	
*CEBPA*	*1050*	F: 5′-AGGAGGATGAAGCCAAGCAGCT-3′	113
		R: 3′-AGTGCGCGATCTGGAACTGCAG-5′	
*PPARG*	*5468*	F: 5′-AGCCTGCGAAAGCCTTTTGGTG-3′	129
		R: 3′-GGCTTCACATTCAGCAAACCTGG-5′	
*COL2*	*1280*	F: 5′-CCTGGCAAAGATGGTGAGACAG-3′	127
		R: 3′-CCTGGTTTTCCACCTTCACCTG-5′	
*COL10*	*1300*	F: 5′-CGCTGAACGATACCAAATGCCC-3′	108
		R: 3′-TGGACCAGGAGTACCTTGCTCT-5	

^∗^Annealing temperature 60°C, cycle number 40.

**Table 2:** Among the DEGs between the spheroid and monolayer culture groups, we extracted genes with Log_2_(fold change) ≥ 6, *q* value < 0.05, and FPKM > 50 in the spheroid culture group.

Gene	Spheroid_FPKM	Monolayer_FPKM	Spheroid_vs_monolayer	*q*_value
AREG	200.378	0.263988	9.56804	0.000412899
STC1	403.17	1.17002	8.42871	0.000412899
SLC16A6	79.6871	0.231565	8.42678	0.00660208
CXCL8	616.76	2.14352	8.16859	0.00041289
PTGS2	801.382	2.85902	8.13083	0.00041289
PTHLH	196.705	0.99057	7.63356	0.00041289
FAM20A	53.1832	0.286936	7.5341	0.00142828
NR4A2	79.2241	0.506743	7.28854	0.00041289
APOE	118.754	1.03351	6.84429	0.00041289
IL11	474.824	6.36988	6.21998	0.00041289

## Data Availability

The data that support the findings of this study are available from the corresponding author, M.U., upon reasonable request.
